# Gender Influences on Shoulder Arthroplasty

**DOI:** 10.1007/s12178-021-09737-0

**Published:** 2022-01-04

**Authors:** Nicole J. Hung, Stephanie E. Wong

**Affiliations:** 1 School of Medicine, University of California, San Francisco, San Francisco, CA USA; 2 Department of Orthopaedic Surgery, University of California, San Francisco, 1500 Owens Street, San Francisco, CA 94158 USA

**Keywords:** Total shoulder arthroplasty, Reverse shoulder arthroplasty, Gender, Outcomes, Disparities, Complications

## Abstract

**Purpose of Review:**

As the incidence of shoulder arthroplasty continues to increase, there is growing interest in patient-based factors that may predict outcomes. Based on existing literature demonstrating gender-based disparities following total hip and knee arthroplasty, gender may also influence shoulder arthroplasty. The purpose of this review is to discuss the recent literature on the influence of gender on shoulder arthroplasty, focusing on differences in preoperative parameters, perioperative complications, and postoperative outcomes.

**Recent Findings:**

While both female and male patients generally benefit from shoulder arthroplasty, several differences may exist in preoperative factors, acute perioperative complications, and postoperative outcomes. Preoperatively, female patients undergo shoulder arthroplasty at an older age compared to their male counterparts. They may also have greater levels of preoperative disability and different preoperative expectations. Perioperatively, female patients may be at increased risk of extended length of stay, postoperative thromboembolic events, and blood transfusion. Postoperatively, female patients may achieve lower postoperative functional scores and decreased range of motion compared to male patients. Differences in postoperative functional scores may be influenced by gender-based differences in activities of daily living. Finally, female patients may be at greater risk for periprosthetic fracture and aseptic loosening while male patients appear to be at greater risk for periprosthetic infection and revision surgery.

**Summary:**

Current literature on the influence of gender on shoulder arthroplasty is limited and conflicting. Further research is necessary to delineate how gender affects patients at the pre- and postoperative levels to better inform decision-making and outcomes.

## Introduction

Shoulder arthroplasty is an effective treatment for shoulder pathology including glenohumeral osteoarthritis (OA), rotator cuff arthropathy, and proximal humerus fractures. Since Péan performed the first documented shoulder arthroplasty in 1894, there have been significant advances in implant development and surgical technique [1]. In 1955, Neer developed the first modern total shoulder arthroplasty (TSA) to treat proximal humerus fractures, reporting pain relief in patients who received a proximal humerus prosthesis [2]. There has been a rapid rise in shoulder arthroplasty utilization since 2004, which is partially attributed to the introduction and approval of a reverse total shoulder arthroplasty (RTSA) device by the Food and Drug and Administration (FDA) in November 2003 [3]. RTSA was originally introduced as an effective surgical treatment of rotator cuff arthropathy, but its indications have since expanded to include treatment of proximal humerus fractures, revision shoulder arthroplasty, and tumors [4]. Overall, shoulder arthroplasty is a successful procedure that leads to pain relief and improved function [5•, 6].

Although the exact incidence of glenohumeral OA is unknown, cadaveric and radiographic studies have demonstrated evidence of glenohumeral OA in up to 32.8% of adults over the age of 60 [7, 8]. As the American population continues to age, the incidence of shoulder arthroplasty will continue to increase. Demand for shoulder arthroplasty is estimated to increase by 355–755% by 2030 [3, 9, 10]. In fact, demand for shoulder arthroplasty may overcome that for total hip arthroplasty (THA) and total knee arthroplasty (TKA) [10]. Given the projected increase in shoulder arthroplasty, it is important to consider patient factors that may impact postoperative outcomes.

Previous studies have described disparities in outcomes for total hip and knee arthroplasty patients based on gender [11–14]. These differences in outcomes may be impacted by female patients having a worse preoperative functional status before undergoing THA and TKA [15]. Although limited, current literature suggests that gender also influences outcomes for patients undergoing shoulder arthroplasty. The purpose of this review is to discuss the recent literature on the influence of gender on shoulder arthroplasty, focusing on differences in preoperative parameters, perioperative complications, and postoperative outcomes.

## Differences in Preoperative Parameters

Several studies have described differences in preoperative factors between male and female patients undergoing shoulder arthroplasty. These factors include patient age at presentation, preoperative disability level, and preoperative patient expectations. Female patients typically undergo shoulder arthroplasty at an older age compared to male patients [16, 17••]. In a recent study, Okoroha et al. reported an average age at time of TSA and RTSA in female versus male patients of 71.2 ± 8.5 years versus 67.4 ± 8.9 years (*p* < .01) [17••]. Jawa et al. showed a similar trend in patients undergoing TSA as female patients had a mean age of at time of surgery of 66.4 years versus 60.8 years for male patients (*p* = 0.01) [16]. In a study by Wong et al., the mean age of the female cohort undergoing RTSA trended towards being greater than that of the male cohort (70.3 years versus 66.9 years, *p* = 0.078) [18••].

There is mixed evidence on whether female patients undergoing shoulder arthroplasty have greater levels of preoperative disability and lower baseline functional status. In a recent prospective study, Wong et al. found similar baseline range of motion in all planes in female and male patients undergoing RTSA [18••]. Additionally, preoperative 12-Item Short Form Health Survey (SF-12), American Shoulder and Elbow Surgeons Score (ASES), and Visual Analog Score (VAS) pain scores were similar between female and male patients [18••]. Jawa et al. similarly found no difference in preoperative ASES, SF-12, and VAS scores between female and male patients undergoing TSA.

In contrast, Okoroha et al. recently reported significantly lower preoperative clinical outcome scores (ASES, Constant, Simple Shoulder Test (SST), *p <* 0.01) in female patients compared to male patients undergoing TSA and RTSA. This study also described decreased range of motion in female patients preoperatively, specifically decreased active abduction (*p* < 0.01), forward flexion (*p* < 0.01), and external rotation (*p* = 0.02) [17••]. In a retrospective review of an international multicenter database of 660 patients undergoing RTSA, Friedman et al. similarly found lower preoperative clinical outcome scores for female patients (SST, ASES, Shoulder Pain and Disability Index (SPADI), and Constant, *p* < 0.001) as well as more limited motion in abduction (*p* = 0.001) and passive external rotation (*p* < 0.001) [19••].

Female and male patients who undergo shoulder arthroplasty appear to have different preoperative expectations. In a prospective study of 63 patients undergoing TSA by Jawa et al., male patients identified participation in sports, sleeping through the night, and maintaining employment as the top three preoperative expectations. On the other hand, female patients selected ability to independently perform household chores and daily routine as the top consideration, which differed significantly from men (*p* < 0.01). Despite this difference, female patients also valued sleeping through the night and exercising/participating in sports [16]. According to Henn et al., additional preoperative expectations that were more important to female patients than male patients undergoing TSA included stopping further shoulder dislocation (*p* < 0.05), improving psychological well-being (*p* < 0.05), and improving driving ability or ability to put on a seatbelt (*p* < 0.05) [20]. Additionally, Mancuso et al. previously described improvement in ability to provide self-care and complete pain relief as preoperative expectations of female patients undergoing shoulder surgery [21].

## Differences in Acute Perioperative Outcomes

Existing literature suggests that female patients are at an increased risk of prolonged hospitalization after undergoing shoulder arthroplasty; however, recent studies challenge these findings [22–25]. Through large national database analysis, Menendez et al. found that female sex is associated with extended length of stay following RTSA (OR = 1.26, *p* = 0.001) and TSA (OR = 2.14, *p* < 0.001) [25]. Dunn et al. reported similar findings with male patients requiring shorter length of stay after TSA (OR, 0.44 [95% CI: 0.29, 0.66]; *p* < 0.0001) [24]. Additionally, Matsen et al. analyzed 17,311 patients from the New York Statewide Planning and Research database and found that female sex is associated with longer hospitalization for all shoulder arthroplasty procedures including hemiarthroplasty, TSA, and RTSA [26].

In contrast to these studies, Okoroha et al. did not find an association between female sex and increased length of stay following shoulder arthroplasty [17••]. Wong et al. similarly found no difference in length of stay between female and male patients after RTSA (men, 2.32 days; women, 2.58 days; *p* = 0.18) [18••]. Possible explanations for the differences in these findings include the confounding effect of study demographic (Matsen had 63% women and Wong had 51.3% women) and different types of arthroplasty included in the different analyses. Furthermore, 23.6% of shoulder arthroplasty in the Matsen study was performed for fracture, which was the diagnosis associated with the longest hospital stay [26].

Additional perioperative complications to consider include thromboembolic events, blood transfusions, short-term readmission, and opioid use. While female patients do not appear to be at increased risk of short-term readmission or increased inpatient opioid use after shoulder arthroplasty, there is an association between female sex and increased risk of thromboembolic events and blood transfusion after shoulder arthroplasty [18, 22, 26, 27]. Matsen et al. found no differences in risk of 90-day readmission between female and male patients after hemiarthroplasty, TSA, and RTSA [26]. Wong et al. found no difference in postoperative inpatient opioid use between female and male patients after RTSA [18••]. In a prospective study including 3480 patients undergoing TSA, Singh et al. demonstrated that female gender is associated with a significantly higher risk of 90-day thromboembolic event after TSA (*p* < 0.03) [22]. Finally, Gruson found female sex to be an independent risk factor for blood transfusion (OR 2.22; 95% CI, 1.03–5.81; *p* = 0.04) [26].

## Differences in Postoperative Outcomes

Although female and male patients both appear to benefit from shoulder arthroplasty in pain relief and ROM, disparities in postoperative functional outcomes may exist [16–19, 28]. Okohora et al. showed no difference in postoperative satisfaction between female and male patients after shoulder arthroplasty with 90% of each cohort reporting to be “better” or “much better” postoperatively. Additionally, both cohorts showed significant postoperative improvement in all outcome measures. Yet, compared to male patients undergoing shoulder arthroplasty, female patients started and ended with lower outcome scores and range of motion [17••]. Furthermore, male sex has been associated with better outcome scores and ROM for all measurements except external rotation after RTSA [19••].

In contrast, Wong et al. found comparable improvements in pain and ROM after RTSA for male and female patients; however, male patients had higher postoperative ASES function and SF-12 PCS scores at 1- and 2-year follow-up. For SF-12 PCS scores, female patients only improved 69% (1-year follow-up) and 42% (2-year follow-up) as much as the male patients. A similar trend in improvement was noted with ASES function scores at 1- (94%) and 2-year (75%) follow-up [18••].

Findings from several older studies also suggest worse postoperative functional outcome in female patients [28, 29]. Donigan et al. showed that female patients had lower SST scores postoperatively compared to male patients after TSA (*p* = 0.0001); however, this finding may be confounded by the inclusion of hemiarthroplasties and patients with rheumatoid arthritis [16, 28]. Matsen et al. found that the strongest correlates with postoperative shoulder function were male gender (*p* < 0.0001), social function (*p* < 0.0001), mental health (*p* < 0.0001), and preoperative shoulder function (*p* < 0.0001) [29]. Contrary to these findings, Jawa et al. found no differences between men and women in ASES/SF-12 at 3-year follow-up after TSA [16].

Differences between female and male patients after undergoing shoulder arthroplasty may also exist in rates of implant-related failure, periprosthetic fracture, and periprosthetic infection. Okoroha et al. found that female patients were more likely to experience implant loosening (*p* = 0.03) and periprosthetic fracture (*p* = 0.01) while male patients had a higher incidence of periprosthetic infection (*p* < 0.01). However, the overall complication rate between the two cohorts was similar (3.2% female vs. 3.6% male, *p* = 0.61) [17••].

Male patients may be at higher risk of revision after shoulder arthroplasty. An older German registry study showed that male sex was associated with a higher revision burden compared to that of female patients following TSA [30]. Similarly, in an analysis of factors for revision TSA using the Mayo Clinic Total Joint Registry, Singh et al. found male sex to be significantly independently associated with increased risk of revision (HR 1.72 (1.28,2.31), *p* < 0.001) [31]. Contrary to these studies, Matsen et al. did not find male sex to be associated with an increased risk of revision following TSA and hemiarthroplasty; however, an analysis was not stratified with respect to sex for the different procedures [26].

## Conclusions

Both female and male patients typically benefit from shoulder arthroplasty. Yet, differences in preoperative factors, acute perioperative complications, and postoperative outcomes may exist. Female patients typically undergo shoulder arthroplasty at an older age compared to male patients. They may also have worse preoperative disability and different preoperative expectations compared to male patients undergoing shoulder arthroplasty. Female patients may place greater importance on performing ADLs and household chores compared to male patients when considering preoperative expectations.

Age, preoperative disability, and preoperative expectations are likely interconnected. Age has been shown to influence preoperative expectations and worsen preoperative disability levels [20, 21]. Typically, younger patients place higher value on participation in sports, contributing to differences observed in preoperative expectations. Furthermore, the question of why female patients present at a later age may be related to differences in utilization of joint replacement between female and male patients. Although conflicting, existing literature suggests patient willingness to undergo surgery may influence utilization rates [32, 33]. Qualitative studies have shown that female patients with hip and knee OA are often more accepting of greater functional decline over time compared to their male counterparts. Additionally, female patients may be less accepting of disrupting their roles and responsibilities as family caregivers to undergo surgery [32]. Another study cited increased concern of female patients about postoperative recovery as a contributor to decreased utilization of TKA [33]. On the other hand, in a survey of 48,218 patients evaluating willingness to undergo total knee arthroplasty (TKA) and total hip arthroplasty (THA) (response rate of 72%), Hawker et al. found that female patients were three times less likely to undergo TKA/THA after controlling for willingness to undergo surgery [11]. These considerations may be similar in female patients who are contemplating shoulder arthroplasty, which could lead to later presentation and lower utilization rates.

In terms of acute perioperative complications, older studies suggest that female patients have increased length of stay following shoulder arthroplasty, but findings from more recent studies contradict this trend. These differences may be due to confounding factors such as demographics and inclusion criteria of the older studies. For example, in Matsen’s study, hemiarthroplasty was included in procedure types, and proximal humerus fractures constituted a greater percentage of surgical indications; this diagnosis was independently associated with increased length of stay [26]. Additionally, female patients appear to be at an increased risk of blood transfusion and acute thromboembolic events whereas readmission and inpatient opioid use rates between female and male patients are comparable. This trend reflects previous findings’ robust data regarding increased bleeding risk and transfusion rate in female patients undergoing elective procedures [34].

While both female and male patients benefit from shoulder arthroplasty similarly in pain score improvement, there are apparent differences in ROM improvement and postoperative functional scores. Existing literature has shown a trend towards lower postoperative functional scores and possibly less improvement in ROM postoperatively in females. However, a recent prospective study contradicted these findings, reporting similar postoperative ASES and SF-12 scores after TSA [16]. As Wong et al. noted, when considering the reason for apparently lower functional scores in females despite similar improvement in pain scores, it is important to consider how female patients may experience greater functional impairment based on differences in activities of daily living (ADLs). For example, brushing longer hair requires external rotation and donning a bra requires more internal rotation. Inability to perform these tasks may lead to lower functional scores [18••]. Based on this assessment, Lynch proposed that future research should include a formal analysis across genders of reliability, responsiveness, variability, and validity of commonly used patient-reported outcome measures [35•]. This poses the question of whether a new validated means of measurement is potentially needed given differences in ADLs between female and male patients. Finally, we should consider how improvements in these outcome scores translate clinically between male and female patients. Simovitch et al. found that female patients required a lower minimally clinically important difference (MCID) in all outcome metrics and motion measurements except for the SPADI score after TSA [5•].

Female and male patients appear to be at risk of different postoperative complications, although the overall complication rate seems to be comparable. Female patients may be more likely to experience implant loosening and periprosthetic fracture while male patients may be more likely to sustain periprosthetic infection and undergo revision surgery. Okoroha et al. hypothesized that this difference is partially explained by increased fall rate in female patients leading to mechanical complications [17••].

The current literature on gender-based differences in shoulder arthroplasty is limited in scope and often conflicting in conclusions. Further research is needed to comprehensively evaluate the influence of gender on shoulder arthroplasty, especially as the incidence of shoulder arthroplasty continues to increase. With a deeper understanding of how gender specifically impacts patients undergoing shoulder arthroplasty at the preoperative and postoperative levels, we can better inform patient expectations and decision-making, and ultimately improve patient outcomes.
